# The role of biological sex in neurophysiological associations of patients with chronic osteoarthritis pain: a prospective cross-sectional study

**DOI:** 10.1016/j.bjane.2025.844639

**Published:** 2025-05-16

**Authors:** Kevin Pacheco-Barrios, Marcel Simis, Paulo S. de Melo, Ingrid Rebello-Sanchez, Karen Vasquez-Avila, Sara Barbosa Franco, Paola Gonzalez-Mego, Linamara Battistella, Marta Imamura, Felipe Fregni

**Affiliations:** a Neuromodulation Center and Center for Clinical Research Learning, Harvard Medical School, Spaulding Rehabilitation Hospital and Massachusetts General Hospital, Boston, USA; b Universidad San Ignacio de Loyola, Vicerrectorado de Investigación, Unidad de Investigación para la Generación y Síntesis de Evidencias en Salud, Lima, Peru; c Universidade de São Paulo, Faculdade de Medicina, Hospital das Clinicas (HCFMUSP), São Paulo, SP, Brazil

**Keywords:** Chronic pain, Confounding variable, Osteoarthritis, Pain, Sex

## Abstract

**Background:**

This study aims to explore the role of sex as a confounder and effect modifier in the associations of clinical outcomes, pain-related outcomes, and neurophysiological measurements in chronic knee OA pain subjects.

**Methods:**

Sociodemographic, clinical, and neurophysiological data were extracted from 113 knee OA subjects with chronic pain. We performed exploratory multivariate regression models assessing the association of physiological outcomes (Quantitative Sensory Testing [QST], Electroencephalography [EEG], and Transcranial Magnetic Stimulation [TMS]) and clinical characteristics (pain, anxiety, and motor function). In each independent model we tested the role of biological sex as confounder and effect modifier (adding the interaction term).

**Results:**

Females reported higher pain intensity, lower quality of life, diminished pain thresholds, and less EEG alpha power compared to males. Sex negatively confounded the association between pain interference and pain intensity with pain threshold confounding (ranged between -19% to -125%). Moreover, sex acted as an effect modifier, predominantly influencing the relationship between pain interference and frontocentral alpha-delta power in EEG. Similarly, sex modified the association between pain interference and pain threshold. In females EEG and PPT variables explained less variability of pain interference compared to males.

**Conclusions:**

Our study suggests that sex is a confounder and effect modifier mainly in the relationship between neurophysiological variables and pain-related outcomes in a chronic OA pain population. Females may have weaker associations between pain intensity and mechanistic outcomes (EEG and QST). Thus, the use of these biomarkers in females requires further optimization. We therefore reinforce the need for accounting for biological sex in the analysis, not only as a confounder, but as an effect modifier in further randomized trials and observational studies in the field of pain.

## Introduction

Since the National Institutes of Health (NIH) implemented in 1993 a policy to increase the inclusion of women and minorities in clinical trials, many studies have investigated differences between women and men across various domains, including pain perception and experience.^1^, ^2^, ^3^ According to the literature, women have a higher prevalence of chronic pain.^4^ Examples of this epidemiological distribution are disorders such as migraine, fibromyalgia, chronic tension-type headache, irritable bowel syndrome, temporomandibular disorders, interstitial cystitis, low back pain, Osteoarthritis (OA), and other musculoskeletal disorders.^3^^,^^5^, ^6^, ^7^ In OA, females have not only shown higher prevalence but also greater pain sensitivity.^8^^,^^9^ Furthermore, differences between sexes demonstrated a lower pain threshold in women.^10^^,^^11^

Several studies exploring associated factors of chronic pain have found relationships between many clinical (e.g., depression, anxiety, and catastrophizing) and neurophysiological variables (e.g., Electroencephalography [EEG], cortical excitability, and QST) with pain-related variables (pain intensity and interference) in chronic pain populations,^12^, ^13^, ^14^, ^15^, ^16^, ^17^, ^18^, ^19^, ^20^, ^21^, ^22^, ^23^, ^24^, ^25^ however, in some of these studies the full extent of sex as a biological variable to explain residual confounding and individual variability have not been fully explored and this may explain variability across these studies.

Interestingly, even though the relevance of sex for chronic pain studies is well established in the field,^26^ its influence as a confounder and effect modifier in the association of clinical and neurophysiological variables with pain-related outcomes is poorly explored. This might be one of the key explanations for the aforementioned heterogeneity. The lack of controlling and stratification by sex may be introducing bias and misleading the interpretation of studies' results and thus the understanding of the chronic pain phenomenon.

Thus, we aim to explore the influence of sex as a confounder and effect modifier in the associations of pre-specified clinical outcomes (pain catastrophizing, anxiety, depression, and motor function), pain-related outcomes (pain interference), and neurophysiological measurements (such as QST, EEG and cortical excitability) in chronic knee OA pain subjects, oriented by a Direct Acyclic Graph (DAG). We hypothesize that sex will be an important confounder and effect modifier in these associations.

## Methods

### Study design

We performed a cross sectional analysis of chronic knee OA patients from a prospective cohort study “Deficit of Inhibition as a Marker of Neuroplasticity (DEFINE study) in rehabilitation”.^22^ The study protocol and this analysis were approved by the Research and Ethical Committee of Hospital das Clínicas da Faculdade de Medicina da Universidade de São Paulo (HC-FMUSP) **(**Registration number: 86832518.7.0000.0068). This study is in accordance with Brazilian research ethics regulations and the Declaration of Helsinki.

### Study procedures

Patients admitted to the IMREA’s conventional rehabilitation program at the Hospital das Clínicas da Faculdade de Medicina da Universidade de São Paulo (HC FMUSP) were included after signing the informed consent form. The longitudinal cohort (DEFINE Study) included a predetermined sample size of 100 patients.^22^

In a baseline visit, a trained investigator performed clinical and neurophysiological assessments in a standard format. The inclusion criteria were 18 years of age and older, diagnosis of knee osteoarthritis through a clinical and radiological assessment (magnetic resonance imaging or computerized tomography; or bilateral knee radiography), clinical stability verified by medical evaluation, informed consent form signed by the subject, and eligibility criteria met for the Instituto de Medicina Física e Reabilitação (IMREA) rehabilitation program.^22^ The exclusion criteria included patients with any clinical condition that could interfere with their participation in the rehabilitation program, as well as pregnant patients.

### Demographic and clinical assessments

We collected information about the participants from a standardized medical interview. We provide the description of all clinical questionnaires used in this analysis.

### Static and dynamic quantitative sensory testing (QST)

#### Pressure Pain Threshold (PPT)

We defined pressure pain threshold (PPT) as the minimum amount of pressure required to trigger pain in specific regions (thenar, and above the knee – bilateral 1 inch above the patella) using an algometer (kg.cm^-2^).^27^ Three algometry measurements (15-second intervals) were taken to calculate the average. The participants received standardized instructions to verbally express a request to stop the PPT stimulus.

#### Conditioned pain modulation (CPM)

As previous studies,^28^^,^^29^ we used a CPM protocol assessed by changes on PPTs. We asked the subjects to immerse one of their hands into cold water (10°‒12°C) for one minute. After 30-seconds of immersion, the investigator presented the Visual Analogue Scale (VAS) to patients to indicate their pain level regarding the submerged hand. After that, we took three algometric measures (PPTs) for the contralateral hand. After an interval of approximately 10-minutes, the subject immersed the other hand in the recipient, and followed the same protocol.^30^ The CPM response was calculated by the difference between the average baseline PPTs minus the average PPTs during the conditioned stimulus.

### Transcranial magnetic stimulation (TMS)

Cortical excitability was assessed using the Magstim Rapid® stimulator (The Magstim Company Limited, UK). A 70 mm figure-of-eight coil was positioned at a 45-degree angle on the scalp to deliver perpendicular pulses over both the right and left motor cortices for all assessments. The assessor maintained coil stability and orientation manually, without neuronavigation. Muscle responses were recorded through surface electromyography (EMG) using Ag/AgCl electrodes placed on the first dorsal interosseous (FDI) muscle of the hand, with a grounding electrode positioned on the wrist.

The assessment was conducted bilaterally for the upper limb brain region. The motor cortex was localized using the vertex, with a reference point 5 cm towards the tragus. The “hotspot” was identified as the location with the most stable and highest motor evoked potential (MEP) over the FDI muscle.^31^ The resting motor threshold (rMT) was determined as the minimum intensity required to elicit an MEP at the hotspot, with a 50 μV peak-to-peak amplitude in 50% of attempts.^32^ Several parameters were analyzed: 1) MEP amplitude, calculated at 120% of the rMT, measured peak-to-peak; 2) Cortical Silent Period (CSP), the temporary suppression of EMG activity during a sustained voluntary contraction; 3) Short-interval intracortical inhibition (SICI), assessed using a 2 ms interstimulus interval; and, 4) Intracortical facilitation (ICF), which was assessed with a 10 ms interstimulus interval. Ten randomized stimuli were applied for each interval, and the averages were calculated. A bi-hemispheric average of each metric (rMT, CSP, SICI, and ICF) was computed, considering the bi-hemispheric nature of brain perception.^33^ This approach was further justified by the fact that most participants had bilateral knee osteoarthritis. TMS data were recorded and stored for offline analysis.

### Resting-state electroencephalography (EEG)

#### EEG recording

We recorded the EEG following a standardized approach^34^ in a quiet room. Assessors asked the participants to sit comfortably, have their sight directed naturally below the horizon line, not move, or talk, and relax as much as possible. The investigator made sure they did not fall asleep by observing the patients and verbally drawing their attention if drowsiness was noticed. The resting-state EEG was recorded for 5 minutes with eyes closed using a 128-channel EGI system (Electrical Geodesics, Inc) (EGI, Eugene, USA). The EEG was recorded with a band-pass filter of 0.3–200 Hz and digitized at the sampling rate of 250 Hz.

#### Resting-state spectral power analysis

We conducted offline analysis using EEGLab^35^ and MATLAB (R2012a). EEG data were re-referenced to the average and processed using finite impulse response filters: a 1 Hz high-pass filter and a 50 Hz low-pass filter. A blinded assessor manually detected and rejected artifacts, excluding any signals indicating drowsiness or abnormal discharges before the full study (no such discharges were found). Independent Component Analysis (ICA) was then applied, and components associated with artifacts were removed to reconstruct the clean signal.^36^

We used the pop_spectopo function in EEGLab with Fast Fourier Transformation (5-second windows, 50% overlap) to analyze the artifact-free data. Absolute power (μV²) and relative power (specific frequency power/total power from 1 to 40 Hz) were calculated for the following frequency bands: delta (1–4 Hz), theta (4–8 Hz), alpha (8–13 Hz), and beta (13–30 Hz), including sub-bands of low beta (13–20 Hz) and high beta (20–30 Hz). These EEG metrics were computed from three Regions of Interest (ROIs) ‒ central, parietal, and frontal areas ‒ as these regions are crucial for pain perception.^37^ Electrode data from these regions were selected and averaged.

### Outcomes

#### Selection of outcomes

**Figure 1:**
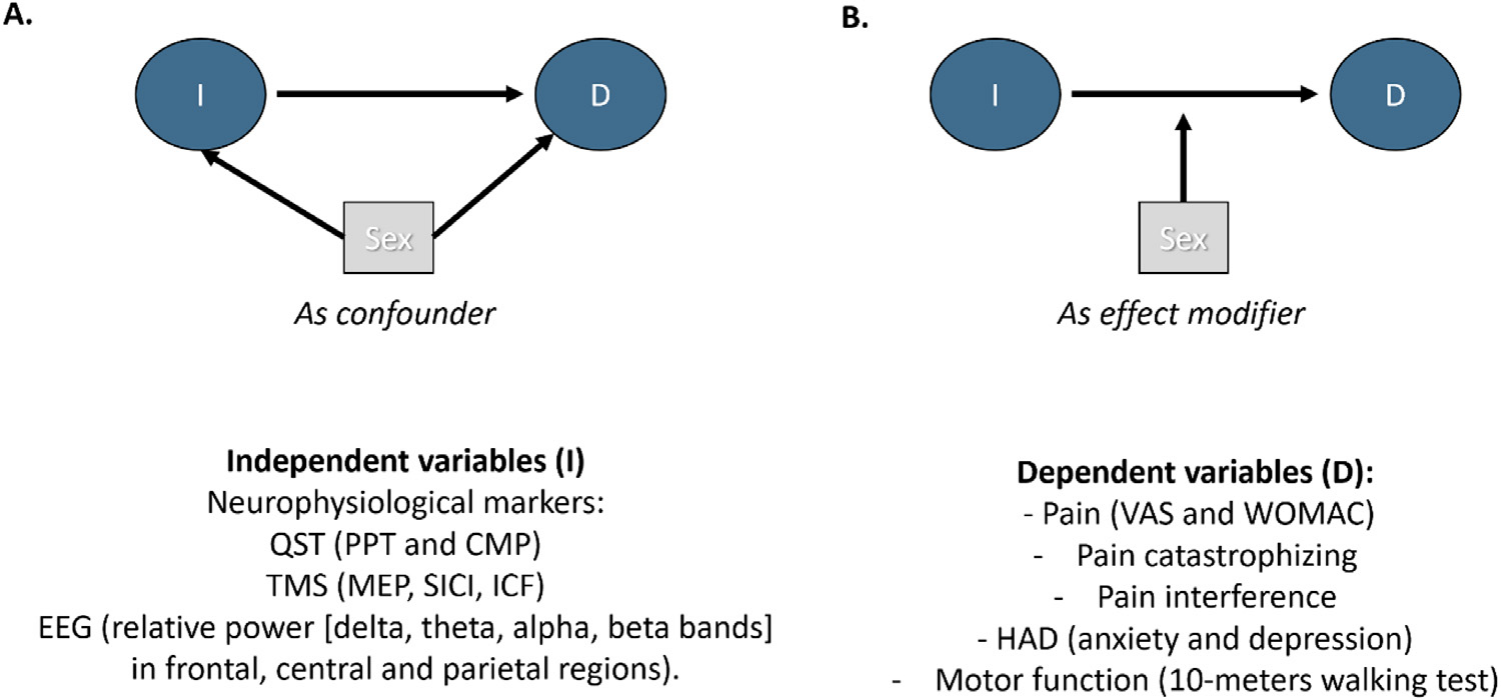
DAG, Direct Acyclic Graph; QST, Quantitative Sensory Tests; PPT, Pain Pressure Threshold; CPM, Conditioned Pain Modulation; TMS, Transcranial Magnetic Stimulation; MEP, Motor-Evoked Potentials; SICI, Short Intracortical Inhibition; ICF, Intracortical Facilitation; EEG, Electroencephalography; VAS, Visual Analog Scale; WOMAC, Western Ontario and McMaster Universities Osteoarthritis Index; HAD, Hospital Anxiety and Depression.

The clinical domains defined as dependent variables were carefully selected from the cohort that generate the data for this analysis.^22^ We decided to include a priori, based on the relevance for the chronic pain field, the classic measures: 1) Pain intensity (Visual Analog Scale – VAS; and Western Ontario and McMaster Universities Osteoarthritis Index – WOMAC – pain scale); pain catastrophizing (Pain Catastrophizing scale); pain interference (sub-scale of the SF-36); anxiety and depression (Hospital Anxiety and Depression scale); and motor function (10-meters walking test). We believe that these variables could provide a consistent and more integrated visualization of the chronic pain profile, since they have shown an influence in how the chronic pain manifests.^38^, ^39^, ^40^ The Direct Acyclic Graph (DAG) with the rationale behind our analysis is provided in Figure 1.

#### Pain

Pain was assessed using the visual analog scale (VAS), the Western Ontario and McMaster Universities Osteoarthritis Index (WOMAC) pain scale (activity-related pain assessment), and the 36-item short form (SF-36). The VAS consists of a 10 cm straight line on a piece of paper. On the beginning is the phrase “no pain” on zero centimeters and on the end “maximum pain” on ten centimeters. We asked patients to mark their discomfort level on the line. Instructions for the patient were “Identify the amount of pain experienced in the past 48h and make a mark perpendicular to the ‘no pain’ – ‘maximum pain’ line”.^41^ Furthermore, the WOMAC pain scale analyzes pain according to 5 items: during walking, using stairs, in bed, sitting or lying, and standing upright. We asked the subject to score the pain in none (0), mild (1), moderate (2), severe (3), and extreme (4).^42^ The pain intensity and interference in the 36-item short form are measured by two questions that respectively ask how much bodily pain the subject had in the past 4 weeks (very severe = 0; none = 100) and how much the pain interfered with normal work (extremely = 0; not at all = 100).^43^

#### Pain interference

We assessed pain interference through the subscale of the 36-item short form (SF-36). The questionnaire consists in eight different domains: 1) physical functioning (10 items); 2) Role limitations due to physical problems (4 items); 3) social functioning (2 items); 4) bodily pain (2 items); 5) general mental health (5 items); 6) role limitations due to emotional problems (3 items); 7) vitality (4 items); and 8) general health perceptions (5 items). Bodily pain is divided into pain intensity and pain interference. The latter consists of the question: “during the past 4 weeks, how much did pain interfere with your normal work (including both work outside the home and housework)?”. The subject could answer this question with one of the options: (100) not at all; (75) a little bit; (50) moderately; (25) quite a bit; (0) extremely. Scores indicate health status in which a lower score correlates to lower health status, and a higher score indicates higher health status.^43^ Pain interference, assessed through the SF-36 bodily pain subscale, was selected as the primary indicator of pain's impact on daily functioning due to its demonstrated reliability with the variables available to use. Moreover, the SF-36 bodily pain (intensity + interference) has demonstrated an area under the curve of 0.679, that is similar to BPI interference, and superior to PROMIS scale. These results conveyed that, besides not being the gold standard, the variable used can provide a degree of reliability.^44^

#### 10-meter walking test

This test evaluates a subject’s time for a short distance walk. We asked the subjects to walk at their normal speed and recommended them to walk 14 meters total so that the initial and final 2 meters were discounted.

#### Hospital Anxiety and Depression Scale (HADS)

This scale consists of 14-multiple-choice questions that quantify symptoms of anxiety and depression. It was divided into two subscales for depression and anxiety separately, each made up of 7 items. The scores for the subscales range from 0 to 21. The scale evaluates the mood during the last seven days.^45^

#### Pain catastrophizing scale

It is a nine components scale judged through a likert of 5 items varying from “almost never” to “almost always” in the extremities. The scale is scored by adding the components and dividing them by the number of answered items. The minimum score is 0 and the maximum 5, with higher scores indicating higher levels of catastrophizing thoughts.^46^^,^^47^

### Statistical analysis

We conducted an exploratory analysis to identify sex as a confounder or effect modifier of the relationship of selected independent variables and pain-related and clinically relevant outcomes (interference, pain catastrophizing, anxiety, depression, and motor function).

Descriptive statistics were used to report baseline variables among males and females. Categorical variables were represented by percentage and absolute values. We represented continuous variables with mean and Standard Deviation (SD). We used histograms and the Shapiro-Wilk to test normality of the variable’s distribution.

To compare males and females, we performed univariate analyses. Categorical variables were tested using the Chi-squared test, while continuous variables were assessed with Student’s t-test. All the variables with a p-value ≤ 0.25 were considered for further exploration. To represent the domains, we were interested in (pain, emotion, and motor function), we performed regressions for the following clinical outcomes (dependent variables): Pain intensity with the visual analogue scale (VAS); WOMAC pain sub score; Pain Catastrophizing Scale (PCS); Pain interference subscale of SF36; Hospital Anxiety and Depression scales; and 10-meter walking test (10MWT). And for the independent variables we included the following neurophysiological markers: QST, TMS, and EEG (Fig. 1).

Additionally, to select relevant associations in which sex could be a confounder or an effect modifier, we performed univariate linear regressions between the independent variables and selected outcomes. All the independent variables with a p-value ≤ 0.25 in this analysis were also considered for further exploration. In the final analysis, we first ran models for the selected outcomes with all the selected independent variables (from the univariate regressions and unbalances between males and females). Subsequently, in each of the models we added sex to assess confounding and the interaction term between sex and the independent variable to assess effect modification. Sex was considered a confounder if it changed the independent variable's coefficient ≥ ± 10% after added in the model. An effect modification was considered to exist when the interaction term was statistically significant (p ≤ 0.05). All the analyses were conducted in STATA 17.0, and the interactions were plotted in R version 1.4.1106.^48^

## Results

Baseline data from the cohort were collected from 113 patients with knee OA pain. In our sample, 19 (16.8%) were males and 94 (83.2%) were females. At the time of assessments on baseline, females had higher pain intensity and pain interference on SF-36 subscales, higher depression and anxiety scores, and lower quality of life as assessed by the SF-36 scale. They also had lower pain thresholds and lower alpha relative power in the central and parietal areas. Noteworthy, females were not different to males regarding motor function and other functionality metrics, other pain intensity scores (VAS and WOMAC), and other neurophysiologic pain-related metrics, such as CPM, rMT, MEP, SICI, ICF, detected by TMS, and EEG frequencies other than alpha. A detailed description of the sample characteristics is provided by sex group in the Table 1.

**Table 1:** Baseline characteristics of chronic knee OA participants by sex groups.

Variables	Male (n = 19)	Female (n = 94)	p-value
Age	67.102 (9.55)	68.9591 (9.45)	0.44
Time of ongoing pain	88.9444 (88.98)	97.1124 (101.01)	0.75
BMI	30.9331 (6.74)	32.1981 (4.99)	0.36
Ethnicity			
White	13 (68%)	59 (63%)	0.35
Black	0 (0%)	13 (14%)	
Mixed	5 (26%)	17 (18%)	
Asian	1 (5%)	5 (5%)	
Education			
Illiterate	0 (0%)	2 (2%)	0.54
Elementary	6 (32%)	42 (45%)	
High school	8 (42%)	26 (28%)	
Superior	5 (26%)	24 (26%)	
KL	2.16(1.21)	2.49 (1.13)	0.25
Bilateral	17 (100%)	84 (99%)	0.65
Knee replacement	2 (11%)	4 (5%)	0.3
Pain catastrophizing	12.32 (9.78)	14.68 (11.29)	0.4
MOCA	22.63 (4.49)	20.67 (5.09)	0.12
10 meters walking test	10.92 (9.52)	11.81 (6.48)	0.62
TUG	15.09 (8.81)	15.94 (7.63)	0.68
Berg balance scale	49.44 (9.17)	46.97 (10.67)	0.36
Epworth sleepiness scale	10.5 (5.52)	10.14 (6.07)	0.82
VAS pain	5.23 (2.08)	5.59 (2.06)	0.49
Hospital Anxiety and Depression scale			
Depression	2.53 (2.09)	4.59 (3.70)	**0.021**
Anxiety	3.78 (3.08)	6.37(4.35)	**0.016**
SF-36			
Overall	61.97 (20.32)	51.89 (19.59)	**0.046**
Social function	69.74 (29.26)	70.88 (28.91)	0.88
Pain intensity	40 (30.55)	26.82 (20.32)	**0.022**
Pain interference	64.47 (32.61)	46.31 (29.00)	**0.017**
WOMAC			
WOMAC total	46.44 (19.41)	51.71 (19.51)	0.3
Pain	9.89 (4.32)	10.95 (4.16)	0.33
Rigidity	5.17 (1.69)	4.43 (2.12)	0.17
Functionality	31.39 (14.75)	36.34 (14.41)	0.19
QST			
PPTs ‒ Knee	7.96 (3.32)	4.19 (1.72)	**< 0.001**
PPTs ‒ Upper limb	7.62 (2.07)	5.32 (1.78)	**< 0.001**
CPM	0.95 (1.34)	1.02 (1.29)	0.84
TMS			
Motor threshold	53.61 (9.95)	50.91 (11.73)	0.36
Motor evoked potential	2.11 (2.45)	1.75 (1.10)	0.33
SICI	0.58 (0.41)	0.45 (0.23)	0.074
ICF	1.85 (0.52)	1.61 (0.58)	0.11
CSP	85.99 (36.79)	86.39 (30.51)	0.96
EEG	**Male (n = 5)**	**Female (n = 61)**	
Frontal			
Delta	0.21 (0.13)	0.26 (0.11)	0.3
Theta	0.17 (0.08)	0.22 (0.09)	0.32
Alpha	0.39 (0.15)	0.28 (0.14)	0.075
Beta	0.23 (0.08)	0.25 (0.14)	0.8
Central			
Delta	0.18 (0.14)	0.23 (0.09)	0.27
Theta	0.17 (0.08)	0.22 (0.09)	0.22
Alpha	0.42 (0.18)	0.29 (0.13)	**0.029**
Beta	0.24 (0.11)	0.27 (0.14)	0.62
Parietal			
Delta	0.16 (0.15)	0.21 (0.11)	0.3
Theta	0.14 (0.07)	0.21 (0.09)	0.17
Alpha	0.49 (0.22)	0.33 (0.16)	**0.048**
Beta	0.21 (0.10)	0.25 (0.15)	0.54

### Sex as a confounder

Our results showed that sex appeared to confound the relationship of clinical and neurophysiological variables (Table 2).

**Table 2:** Comparison of models with and without sex on pain outcomes.

	Unadjusted			Adjusted by sex				
	Beta	R^2^	p-value	Beta	R^2^	p-value	ß-coefficients changes (%)	Association direction after adjustment
**Pain interference (SF-36)**								
PPT ‒ Knee	3.069	0.066	0.008	2.493	0.071	0.074	-19%	Decreased
**VAS Pain**								
PPT ‒ Knee	-0.211	0.065	0.008	-0.624	0.074	0.006	+196%	Increased
ICF	-0.785	0.048	0.025	0.195	0.049	0.032	-125%	Decreased and inverted
**WOMAC pain**								
PPT ‒ Knee	-0.545	0.118	< 0.001	-0.638	0.126	< 0.001	-17%	Decreased and inverted
Central theta	-14.913	0.113	0.006	-16.662	0.182	0.002	+12%	Increased
Parietal theta	-11.800	0.082	0.021	-13.659	0.150	0.007	+16%	Increased
**10 meters walking test**								
PPT ‒ Knee	-0.862	0.097	0.001	-1.123	0.116	< 0.001	+30%	Increased
**HAD ‒ Anxiety**								
Central theta	-10.929	0.061	0.045	-12.037	0.088	0.028	+10%	Increased

#### Sex as negative confounder

Female sex negatively confounded the associations between pain-related outcomes (Pain interference and WOMAC Pain) and PPT; as well as the association of intracortical facilitation and pain intensity (VAS). Moreover, the relationship of pain interference was stronger when not controlled for female sex. The magnitude of confounding ranged between -19% and -125% of changes in the beta coefficients (Table 2).

#### Sex as positive confounder

On the other hand, some associations became stronger if controlled for female sex such as the association of VAS pain intensity and PPT (+196% of beta coefficient changes) and the relationship between the 10 meters walking test and PPT. Furthermore, sex positively confounded the relationship between pain intensity (WOMAC) and anxiety with EEG power (central theta and parietal theta). The magnitude of confounding ranged between +10% and +196% of changes in the beta coefficients. The details of the models and the coefficients’ ratios are described in Table 2.

### Sex as an effect modifier

The role of sex as an effect modifier was explored in the previous models by adding the interaction term.

Sex was an effect modifier of the relationship between pain interference and PPT, alpha relative power (frontal and parietal), delta relative power (frontal and central), and theta relative power (central and parietal). Further details are described in Table 3.

**Table 3:** Model interactions with sex in pain outcomes.

	Beta	R^2^	p-value interaction
**Pain interference (SF-36)**			
PPT ‒ upper limb	-8.579	0.092	0.028
Frontal alpha	219.965	0.162	0.005
Parietal alpha	115.776	0.115	0.034
Frontal delta	-269.196	0.176	0.003
Central delta	-233.989	0.161	0.006
Central theta	-443.843	0.091	0.031
Parietal theta	-476.197	0.0j92	0.024
**10 meters walking test**			
PPT ‒ upper limb	1.996387	0.1110	0.024

**Figure 2:**
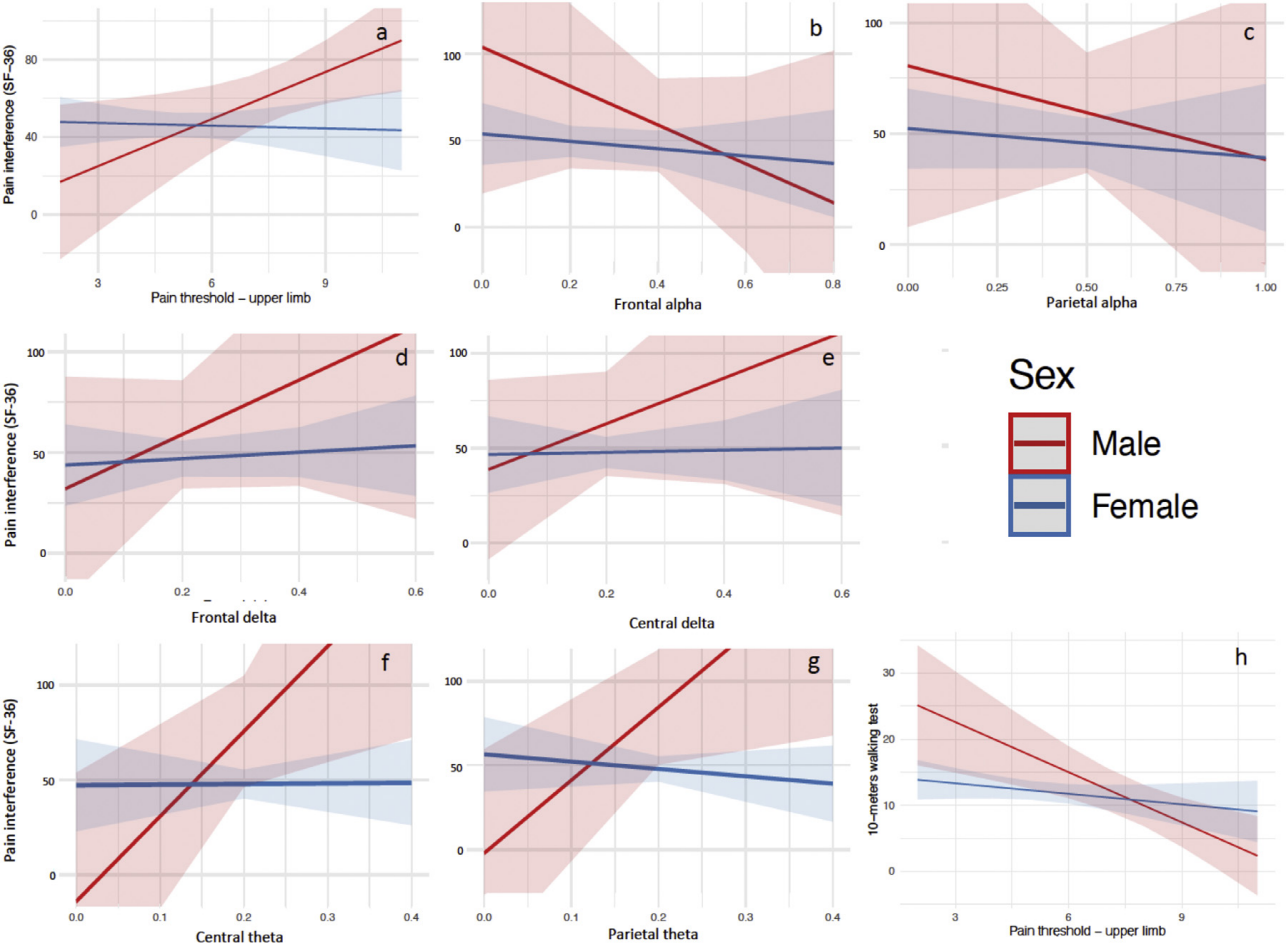
Effect modification by sex.

Interestingly, sex modifies the effect of PPT in predicting pain interference on SF-36, with women having a weaker correlation with PPT in comparison to men, as shown by their flatter graphical representation (Fig. 2). This pattern is seen for all the plotted interactions for which there was an effect modification, including with motor function outcome (10MWT). We did not see effect modifications with sex and other independent variables for emotion outcomes or for WOMAC pain and VAS (Fig. 2).

## Discussion

### Main findings

In our analysis, the outcomes of almost all the domains had their relationships with covariates confounded by sex. In those cases, sex was frequently a confounder of clinical covariates, especially pain interference.

On the contrary, sex as an effect modifier affected mostly the relationship of neurophysiological variables (EEG covariates, TMS assessments, and static QST [Pain threshold]) with pain-related outcomes (SF-36 pain intensity and interference). PPT was the independent variable most frequently confounded and modified by sex. In these circumstances, females were consistently the stratum in which the relationships were weaker, as we can see by their smaller slopes in the graphs (Fig. 2). In fact, in some cases the female slope is almost nonexistent, fitting a horizontal line (Fig. 2 a, e, f). Given the relationship between pain-related outcomes and mechanistic variables is weaker in females, it is likely that other factors may play more important roles in the way that females perceive pain.

### Sex and neurophysiological assessments

Indeed, the extent to which pain can be quantitatively assessed is unknown.^49^ In that way, there has been an attempt to identify more mechanistic ways to evaluate of pain, including several neurophysiological assessments like EEG and EMG studies, neuroimaging, quantitative sensory testing, and genetics.^50^ Currently, many researchers investigate Quantitative Sensory Testing (QST) as a way to objectively assess pain and as a potential reliable predictor or biomarker of pain chronification,^51^ treatment response,^52^ and even as a diagnostic tool.^53^

However, these studies have shown heterogeneous results,^54^ and not accounting for sex in the analysis might be one explanation. It is surprising to realize that even though the role of sex is well settled in pain literature, studies still neglect the potential sample that could arise from not properly accounting for sex in design planning and statistical analysis.^55^ Our findings strongly suggest sex should be accounted for not only as a confounder but as an effect modifier in chronic pain related analysis.

Flingeton et al. conducted a systematic review and meta-analysis assessing differences in QST results between OA and control populations.^56^ They found a high level of heterogeneity (I = 82%, p < 0.001) that could not be explained by variations in testing sites. Interestingly, five out of the fifteen observational studies included did not adjust for sex in the analysis; one study only included females in the trial; and the percentage of females in OA groups varied significantly from 24% to 81.25% among the remaining studies. None of them addressed sex as an effect modifier. Still, no subgroup analysis by sex was performed in the meta-analysis.

Our results are similar for the study of cortical excitability, as the same reasoning can be applied to the association of TMS and EEG findings with pain-related outcomes. Previous research has evaluated the influence of cortical excitability in chronic pain populations. Some of them have found associations,^21^ while others did not convey the same results.^57^ Similarly, some EEG studies have detected patterns and associations in chronic pain populations, while others did not show similar results.^20^ The struggle to define the real association of these factors and their role in chronic pain may be led by the lack of addressing sex effects in these variables.

Our study demonstrated that females have a lower influence of cortical silent period (as a representation of cortical inhibition drove by GABA-B pathways) and alpha, delta, and theta oscillations in pain. Therefore, the inclusion of males and females in the same analysis to find associations of these neurophysiological variables and clinical chronic pain outcomes (as pain intensity, motor function, and emotional measures) may lead to heterogeneous results depending on the characteristics of the samples and the strength of the associations in males, but not in females.

The current study has shown that less alpha power in the frontal and parietal regions may have a relationship with the pain process which seems to be different in females and males, and another study has also shown differences between females and men with neuropathic pain regarding alpha frequency oscillations.^58^ It may lead more studies to assess more men in chronic pain studies, once it is already known that women were more frequently assessed than men in general^20^ mainly because of the epidemiological rates of pain in females. Especially for EEG biomarker development and neurofeedback protocols, where gender differences have been noticed,^59^ it is important to consider sex aspects in the analysis.

### Potential explanations for sex influences in chronic pain

#### Biological factors

Several observational studies have shed light to the presence of intrinsic biological differences in nociceptive response between males and females, while gonadal hormones are assumed to be the main underlying pathway. Reports of pain symptomatology following patterns according to women’s menstrual cycles in both healthy and chronic pain female populations^60^, ^61^, ^62^ point out to the direction of physiological causes. Adult literature on experimental pain has consistently shown trends of higher pain sensitivity in females in comparison to males, and among females during the luteal phase.^63^

In that direction, epidemiologic data have revealed that sex differences in chronic pain diseases appear to become more evident in older children around puberty.^64^ A meta-analysis of experimental pain in children and adolescents ranging from 0 to 18 years old found that there were no significant overall differences in cold pain thresholds between boys and girls.^65^ However, subgroup analysis of studies in which the average age was ≥ 12 years depict significant differences (SMD 0.19, 95% CI 0.03 to 0.34; p = 0.02).

It is reasonable to expect gonadal and gonadotrophic hormones to have a direct relationship with nociception, acting directly or indirectly as sensitizing factors in different levels of the peripheral and central nervous system. Nonclinical studies have shown sex steroids as regulators of the endogenous opioid system and adaptive immune system. Modulation of inflammatory markers, peptides, and neurotransmitters intercede in acute pain processes and in the development and maintenance of chronic pain. Certainly, other physiological factors (e.g., genetics) influence and are influenced in this framework, including emotional and cognitive processing.^66^^,^^67^

#### Sociocultural factors

If there were only biological reasons dictating pain perception for different sexes, we would expect them to be reflected in usual neurophysiological correlates and similar in older adults without influence of sex. Our sample was comprised mostly by post-menopause women (mean age ∼69) and post-andropause men (mean age ∼67), so we expected less heterogeneity due to sex hormones. However, sex influences were still found.

Chronic pain is a multidimensional experience that is influenced by biological, psychological, social and cultural factors. Lower socioeconomic status, low levels of education and higher levels of anxiety are associated with higher levels of pain.^68^^,^^69^ Moreover, ethnicity and culture play an important role.

Ethnical differences influence not only pain experiences but also access to healthcare and treatment.^70^ For example, African American and Hispanic White are more likely to report pain than Non-Hispanic Whites^71^ and these minorities receive less adequate treatment for acute and chronic pain compared to their white counterparts.^70^

Another crucial factor is culture. Culture can be understood as the set of rules, norms, practices and believes of a specific group of people and based on it, pain expressiveness, the meaning attributed to pain, the coping mechanisms, and also pain experience differ.^72^ For example, pain expressiveness changes depending on the gender role expectation of the individual, as in some cultures males are supposed to be more stoical (no expression of pain) whereas females have higher pain expressiveness.^72^

It is important to emphasize that sex and gender are not interchangeable words, they are two different concepts that are usually misused in clinical literature. Sex is defined as the genetic biological trait that differentiates men and women whereas, gender refers to the roles, stereotypes, attitudes, norms that an individual identifies with and that the society and culture attributes to them.^73^^,^^74^ Moreover, gender has proven to be an important variable when assessing pain.

A meta-analysis^75^ that assessed the relationship between gender role and experimental pain response showed a positive correlation between masculine and feminine personality traits and pain threshold and tolerance and a negative correlation between gender stereotypes specific to pain and pain threshold and tolerance. In other words, individuals who self-reported higher masculine traits than feminine exhibit higher pain threshold and tolerance. Moreover, the subjects that were more willing to report pain and the ones with higher emotional vulnerability had higher pain intensity scores and unpleasantness.^75^ These findings corroborate the influence of gender in the pain experience and emphasize the need of including this variable, regardless of sex, in pain research.

### Future directions

Since the introduction of health policies to represent more females in research, there has been increasing interest in sex differences in pain.^76^ However, gender is likely to be a more important factor that should be addressed in future research, in addition to the variables measured in our study. Chronic pain has shown to be a biopsychosocial model^77^, ^78^, ^79^, ^80^ thus variables such as gender should be a higher priority in research.^74^

Consequently, sex and, in the future, gender are important variables to be thought of during the whole process of conducting research, from the development of the design to the analysis.

### Limitations

Some limitations were present in our study. The lack of adjustment for multiple comparisons could increase the rate of false positive findings, though, since this is an exploratory analysis and hypothesis-generating finding, we were concerned in reducing the rate of false negatives. Thus, such adjustment is not needed. Confirmatory studies are necessary to confirm the hypothesis arisen here. Moreover, we are aware that SF-36 is not the most accurate scale to assess pain interference, but as this is an important aspect for pain that needs to be evaluated, we decided to use the best available tool to measure it. Also, the unbalance between females and males is another potential limitation justified by the epidemiology of OA, where female cases are more prevalent than males. Future research trying to include more men in the analysis to avoid statistical concerns are needed.

## Conclusion

Although sex has been more commonly addressed as a confounder in observational studies, many still fail to do it, and that is even more prominent in randomized clinical trials. More importantly, effect modification by sex has been neglected in pain studies. Our study demonstrated potential effects of sex, mainly in the relationship neurophysiological variables and pain-related outcomes in a chronic pain population due to knee OA. We, therefore, reinforce the need for accounting for sex in the analysis, not only as a confounder, but as an effect modifier in further randomized trials and observational studies in the pain field. Meta-analyses assessing pooled effect sizes should be vigilant regarding the analysis performed in each of the papers included. In such cases, subgroup analysis by sex might be pertinent. In randomized controlled trials (RCTs), stratified randomization by sex is necessary, while in observational studies analyses should be conducted for sex-based subgroups.

In clinical practice, identifying sex as a potential confounder underscores the importance of tailoring OA rehabilitation interventions to address sex-specific differences in disease presentation and treatment. This finding could be one of the based to development of personalized rehabilitation protocols, such as optimizing physical therapy exercises or customizing pain management strategies, to more effectively meet the unique needs of male and female patients.

## Informed consent

Written informed consent was obtained from the patient(s) for their anonymized information to be published in this article.

## Ethical approval

Ethical approval for this study was obtained from “Faculdade de Medicina da Universidade de São Paulo” (HC FMUSP) (Registration number: 86832518.7.0000.0068).

## Trial registration

Not applicable.

## Guarantor

FF.

## Authors’ contribution

KPB: Conception or design of the work, data analysis and interpretation, critical revision of the article, final approval of the version to be published.

MS: Conception or design of the work, data collection, data analysis and interpretation.

PSM: Data interpretation, drafting the article, critical revision of the article, final approval of the version to be published.

IRS: Data interpretation, drafting the article, critical revision of the article, final approval of the version to be published.

KVA: Drafting the article, critical revision of the article, final approval of the version to be published.

SBF: Data interpretation, drafting the article, critical revision of the article.

PGM: Data interpretation, drafting the article, critical revision of the article, final approval of the version to be published.

LB: Conception or design of the work, data interpretation, critical revision of the article, final approval of the version to be published.

MI: Conception or design of the work, data interpretation, critical revision of the article, final approval of the version to be published.

FF: Conception or design of the work, data interpretation, critical revision of the article, final approval of the version to be published.

## Funding

This study is funded by a grant from “Fundação de Amparo à Pesquisa do Estado de São Paulo” (FAPESP) (SPEC project, fund number 2017/12943-8).

## Conflicts of interest

The authors declare no conflicts of interest.
